# Expression, Polymorphism, and Potential Functional Sites of the *BMPR1A* Gene in the Sheep Horn

**DOI:** 10.3390/genes15030376

**Published:** 2024-03-19

**Authors:** Guoqing Zhang, Mingxing Chu, Hao Yang, Hao Li, Jianxin Shi, Pingjie Feng, Shoufeng Wang, Zhangyuan Pan

**Affiliations:** 1 School of Chemistry and Chemical Engineering, University of Jinan, Jinan 250022, China; zhang_guoq@126.com; 2 State Key Laboratory of Animal Biotech Breeding, Institute of Animal Science, Chinese Academy of Agricultural Sciences (CAAS), Beijing 100193, China; chumingxing@caas.cn (M.C.); yanghao199903318@163.com (H.Y.); haoli0825@163.com (H.L.); shijxovo@163.com (J.S.); fengpingjie0729@163.com (P.F.)

**Keywords:** *BMPR1A*, sheep, horn, allele-specific expressions, SNPs

## Abstract

**Simple Summary:**

This study investigated the relationship between the *BMPR1A* gene and horn type in sheep. The study found that the expression of the *BMPR1A* gene was significantly higher in the normal horn sheep compared to scurred sheep. This trend was observed in multiple sheep breeds. It was also discovered that there is high expression in the skin of three species: cattle, sheep, and pigs. Importantly, it was found that certain nucleotides in the *BMPR1A* gene are likely to be crucial in horn size and horn type.

**Abstract:**

Sheep horns are composed of bone and sheaths, and the *BMPR1A* gene is required for cartilage and osteogenic differentiation. Therefore, the *BMPR1A* gene may have a function related to the sheep horn, but its relationship with the sheep horn remains unclear. In this study, we first utilized RNA sequencing (RNA-seq) data to investigate the expression of the *BMPR1A* gene in different tissues and breeds of sheep. Second, whole-genome sequencing (WGS) data were used to explore the functional sites of the *BMPR1A* gene. Lastly, the allele-specific expression of the *BMPR1A* gene was explored. Our results indicate that *BMPR1A* gene expression is significantly higher in the normal horn groups than in the scurred groups. Importantly, this trend is consistent across several sheep breeds. Therefore, this finding suggests that the *BMPR1A* gene may be related to horn type. A total of 43 Single-Nucleotide Polymorphisms (SNPs) (F-statistics > 0.15) and 10 allele-specific expressions (ASEs) exhibited difference between the large and small horn populations. It is probable that these sites significantly impact the size of sheep horns. Compared to other polled species, we discovered ten amino acid sites that could influence horn presence. By combining RNA-seq and WGS functional loci results, we identified a functional site at position 40574836 on chromosome 25 that is both an SNP and exhibits allele-specific expression. In conclusion, we demonstrated that the *BMPR1A* gene is associated with horn type and identified some important functional sites which can be used as molecular markers in the breeding of sheep horns.

## 1. Introduction

Sheep are multipurpose animals that produce meat, milk, hides, and wool. Their primary function is meat production [1]. Horns can be used as weapons and play an important role in defense against predators and in sexual selection through their use in intra-male competition [2,3,4,5,6]. However, horns may be harmful during sheep farming. Generally, sheep have several horn phenotypes, namely, large normal horns, scurred horns, and no horns. Rams usually have larger horns, and ewes usually have smaller horns. Bruising reduces meat quality, and severe udder injuries, which may be the result of attack by a horned individual, reduce milk yield [7]. Although dehorning is legal in some countries, it can be painful and distressing for animals [8,9]. At the same time, horns are expensive to grow and require resources beyond those needed for maintenance or growth, especially during the winter months when they are a major source of energy expenditure for sheep [10,11]. The study of horn formation and the presence or absence of horns is essential not only for the study of natural and sexual selection but also for breeding to facilitate modern sheep production.

Sheep horns are hollow, paired structures with a skeletal core that are covered by an integument [12,13]. Horn development is thought to be primarily controlled by the skin [14]. During the initial stages of horn bud development, the epidermis ceases to produce hair and begins to synthesize the horn [15]. Once initiated, the primordium of the bony core of the horn forms a separate ossification center in the dermal connective tissue beneath the region of horn formation, which later fuses with the skull. Although no studies have shown that the *BMPR1A* gene is associated with horn formation or horn size, several studies have shown that the *BMPR1A* gene is associated with skeletal development. *BMPR1A* is a protein-coding gene that has previously been reported to contribute to bone development, and *BMPR1A* is required for chondrogenic and osteogenic differentiation [16,17,18]. Studies have shown that the *BMPR1A* gene regulates chondrogenic lineage differentiation and endochondral bone formation through its effects on chondroblast proliferation and chondrocyte differentiation [19,20]. Based on our previous study, we screened the genes that were differentially expressed in the small-horn and large-horn groups. GO (Gene Ontology) enrichment analysis of these genes revealed that the *BMPR1A* gene is involved in genes associated with epithelial cell proliferation and cartilage development. We therefore hypothesize that the *BMPR1A* gene is likely to be involved in the formation of the sheep horn. In this study, we first analyzed the expression characteristics of *BMPR1A* in different tissues of sheep using RNA-seq data to understand the basic function of *BMPR1A*. Then, we analyzed the horn-related functional sites in *BMPR1A* using WGS data. This study will provide some valuable molecular makers in sheep horn breeding.

## 2. Materials and Methods

### 2.1. Animal and Sample Collection

All the experimental procedures mentioned in the present study were approved by the Science Research Department (in charge of animal welfare issues) of the Institute of Animal Sciences, Chinese Academy of Agricultural Sciences (IAS-CAAS) (Beijing, China). Ethical approval on animal survival was given by the animal ethics committee of IAS-CAAS (No. IASCAAS-AE-03, 12 December 2016). This study used RNA-sequencing (RNA-seq) data previously collected by our laboratory (PRJNA1003277) [21]. A total of fifteen Tibetan sheep samples were collected in Dangxiong, Tibet, China. These Tibetan sheep are all female and aged between 2 and 4.5 years old. Our Tibetan sheep samples were divided into two groups: one group consisted of 7 sheep with scurred horns (0–12 cm), while the other group consisted of 8 sheep with normal horns (>12 cm) (Supplementary Table S1). The soft-horned tissue was collected from these 15 sheep, placed in deep cryopreservation tubes, and stored in liquid nitrogen.

### 2.2. RNA Sequencing Data Filtering, Comparison, Assembly, and Processing

RNA sequencing (RNA-seq) data consisted of publicly available and laboratory-collected data. We obtained 2915 high-quality sheep RNA-seq data samples from the National Center for Biotechnology Information (NCBI) database (https://www.ncbi.nlm.nih.gov/, accessed on 15 March 2023) and EBI (https://www.ebi.ac.uk/, accessed on 15 March 2023), which are publicly available. Both the public data and the laboratory-collected data underwent the following processing. Raw data with contaminating adapter molecules, reads containing ploy(N), and low-quality reads were removed using Trim Galore (v.0.6.7). The “--chimSegmentMin 10” and “--outFilterMismatchNmax 3” parameters of the STAR (v.2.7.7a) software were used to align clean reads with the sheep reference genome (the ARS-UI_Ramb_v2.0) [22]. Eventually, high-quality RNA-seq clean datasets were obtained for subsequent analysis, with unique mapping reads > 85% and the number of clean reads > 20,000,000. The expression levels of genes were normalized by calculating the number of transcripts per kilobase fragment (FPKM) per million mapped transcripts and the exonic model by the number of transcripts per kilobase (TPM) per million exons using the prepDE.py script of Stringtie (v.2.1.5) [23]. The raw counts of these genes were subsequently extracted using featureCounts (v.2.0.1) [24].

### 2.3. Expression of the BMPR1A Gene in Normal Horn and Scurred Groups

To investigate the difference between *BMPR1A* in normal horn and scurred groups, we produced a boxplot using ggplot2 (v.3.4.0) in R (v.4.2.3). To further understand the distinctions in the expression of *BMPR1A* exons between the two groups, we utilized the dexseq_prepare_annotation2.py script of Subread_to_DEXSeq (https://github.com/vivekbhr/Subread_to_DEXSeq, accessed on 21 April 2023) to format the genome annotation (GTF) file. Following this, we processed the formatted GTF file and the counts matrix output by featureCounts using load_SubreadOutput.R in Rstudio and constructed the DEXSeqDataSetFromFeatureCounts (dds) object. Finally, we performed an exon difference analysis.

### 2.4. Analysis of the Expression Profile of the BMPR1A Gene

To explore *BMPR1A* gene expression across species, we downloaded publicly available RNA-seq data for sheep, pigs, cows, and humans. Additionally, RNA-seq data for 2651 pigs were obtained from the GTEx project, and TPM gene expression values were acquired from http://piggtex.farmgtex.org/ (accessed on 10 September 2023). Furthermore, RNA-seq data for 4359 cows were obtained from the GTEx project, whereas bovine gene expression values in transcripts per million were acquired from https://cgtex.roslin.ed.ac.uk/ (accessed on 1 October 2023) [25]. We downloaded TPM values for genes in the RNA-seq data of 9810 human samples from https://gtexportal.org/home/datasets (accessed on 5 August 2023) [26,27]. Sheep, pig, cow, and human RNA-seq data were pooled to arrange tissue samples, which were subsequently classified into 16 distinct tissues. The average TPM values for the *BMPR1A* gene were calculated for each species (Supplementary Table S2). To further investigate gender and tissue-specific expression variances in the *BMPR1A* gene of sheep, we analyzed RNA-seq data. We collected information on sheep breeds from publicly available RNA-seq data and excluded breeds with small sample sizes. The final 8 breeds of sheep obtained were Bashiba, Chinese Merino, Hu, Minxian Black Fur, Spanish Churra, Tan, Texel, and Tibetan. The sheep were divided into three subgroups: polled, scurred, and normal.

### 2.5. Analysis and Three-Dimensional Structure Prediction of the *BMPR1A* Protein

To investigate disparities in the BMPR1A protein across various species, we retrieved the FASTa files for this protein from NCBI for 18 different species, including sheep, goat, and cattle. Using MEGA11 software, we produced a protein evolution tree for BMPR1A [28]. The default parameters of the ClustalW method were used for alignment [29,30]. The maximum likelihood method was used to develop a circular evolutionary tree for BMPR1A proteins. To determine whether amino acids are located at key positions in the protein, we used the default parameters of AlphaFold2 to predict the 3D structure of the protein BMPR1A protein [31]. 

### 2.6. Whole-Genome Sequencing Analysis

Whole-genome sequencing (WGS) data consisted of publicly available and laboratory-collected data. The publicly available data for 3125 sheep were downloaded from NCBI, including PRJNA304478, PRJNA325682, PRJNA479525, PRJNA624020, PRJNA675420, PRJNA822017, PRJNA30931, PRJNA480684, PRJNA509694, PRJNA779188, and PRJNA783661 [32,33,34,35,36,37,38,39,40,41,42,43]. The sample of 3125 sheep was divided into horned and hornless sheep. F-statistic (Fst) values were calculated using vcftools (v.0.1.16) to discriminate SNP loci that were significantly different in these sheep populations. Small Tail Han sheep were utilized as the laboratory breed, and horn length was quantified for 38 individuals from the root to the outermost end of the horns. The length of the sheep’s right horn was used to determine the length of the sheep’s horn. Trimmomatic (v.0.39) was used to trim the sequencing reads, and FastQC (v. 0.12.1) was used to assess the quality of the raw sequencing data [44]. The qualified reads were then mapped, sorted, and deduped to the sheep reference genome by BWA (v.0.7.17) and Picard (v. 3.1.1) [45,46]. We used the default parameters of the pipeline in the genome analysis toolkit (GATK) (v.4.2.5.0) process to predict the SNP site [47,48]. We used SnpEff (v.4.3) to annotate the mutation information [49]. To determine the relationship between horn length and different genotypes, the dominant model was used. We conducted a deeper analysis of the SNP chain by applying the --maf 0.45 --min-meanDP 5 filters in vcftools (v.0.1.16) and then used LDBlockShow (v.1.40) to identify the chains of SNP loci [50,51].

## 3. Results

### 3.1. The Expression of the BMPR1A Gene in Sheep with Different Horn Types

**Figure 1:**
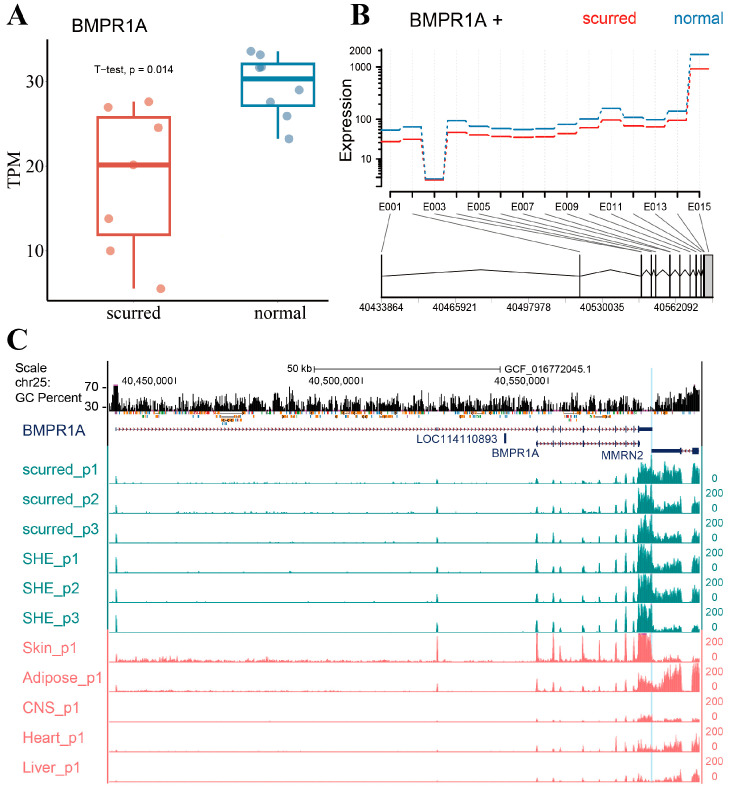
(**A**) Differential expression of *BMPR1A* in scurred and normal horn group groups. (**B**) Expression of *BMPR1A* exons in scurred and normal horn groups. Expression refers to the fitted expression estimates from the GLM regression, and E001–E015 represents the number of exons. The red line represents the scurred group, whereas the blue line represents the normal horn group. (**C**) Differential expression of the *BMPR1A* gene in horn tissues and other tissues. The region labeled in blue represents the overlap between the *BMPR1A* and *MMRN2* genes.

The *BMPR1A* gene is located on chromosome 25 and has two transcripts: XM_012104920.4 and NM_001280714.1. We use transcript XM_012104920.4 to represent the *BMPR1A* gene in this paper. As shown in Figure 1A, the expression of the *BMPR1A* gene was significantly higher in the normal horn group than in the scurred group (*p*-value = 0.014). The exons of the *BMPR1A* gene are predominantly located in the 3′ end of the gene. As shown in Figure 1B, all exons of *BMPR1A* showed higher expression in the normal horn group. We specifically checked the gene expression, GC percent, and repeats of the *BMPR1A* gene. As shown in Figure 1C, all exons were expressed in both the scurred and normal horn group. However, the expression of exons greatly varied in different tissues. Skin and adipose tissue showed a similar expression pattern to the Exon 13 region of the *BMPR1A* gene in the sheep horn. Expression of exons except for the Exon 1 region was higher in skin tissues than in other tissues. Additionally, it was observed that Exon 1 and Exon 2 regions were nearly not expressed in the central nervous system (CNS), heart, or liver tissues. Moreover, the expression of various other exon regions was lower in these three tissues compared with skin and horn tissues. Interestingly, we observed that the *BMPR1A* gene shares a region with the *MMRN2* gene. We hypothesize that this region is likely a component of the *BMPR1A* gene. 

### 3.2. The Tissue-Specific Function of the BMPR1A Gene in Sheep

**Figure 2:**
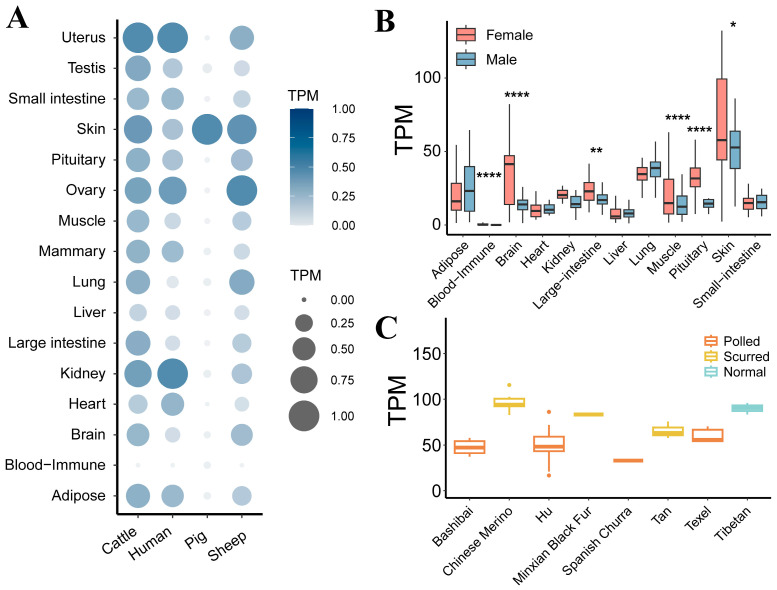
(**A**) Expression of *BMPR1A* in various tissues of four species. Darker colors and larger dots indicate higher TPM values for the *BMPR1A* gene in this tissue. (**B**) Sex differences in the expression of *BMPR1A* were observed in various tissues of sheep. Asterisks indicate the *t*-test *p*-value: * indicates *p*-value < 0.05, ** indicates *p*-value < 0.005, **** indicates *p*-value < 0.00005. (**C**) Expression of *BMPR1A* in different sheep breeds.

To better understand whether there exists a difference in *BMPR1A* gene expression among normal horn and scurred groups in different sheep breeds, we generated Figure 2. Figure 2A displays *BMPR1A* gene expression across 16 tissues in four different species and shows that the *BMPR1A* gene exhibited high expression levels in three species: sheep, cattle, and pigs. The *BMPR1A* gene exhibited the second-highest level of expression in sheep and bovine skin, but its expression in porcine skin had the highest TPM level and was much higher than in other tissues. Additional studies were performed to determine if there were sex differences in the expression of the *BMPR1A* gene in different sheep tissues. As depicted in Figure 2B, significant differences in *BMPR1A* gene expression were observed among the skin, brain, colon, muscle, blood–immune, and pituitary tissues (*p*-value < 0.05). In all six tissues displaying significant differences, *BMPR1A* expression was higher in ewes than rams. We investigated whether the expression of the *BMPR1A* gene remained consistent across various sheep breeds, as depicted in Figure 2C. The expression of the *BMPR1A* gene was lower in all four breeds of hornless sheep (Bashibai, Hu, Spanish Churra, and Texel) compared with Chinese Merino, Minxian Black Fur, and Tan sheep and the three breeds of scurred sheep. The above results suggest high expression of the *BMPR1A* gene in skin tissues across several species, with a sex-specific difference in expression. Additionally, there was variation in expression observed between polled and scurred sheep.

### 3.3. The Potential Function of BMPR1A Protein

**Figure 3:**
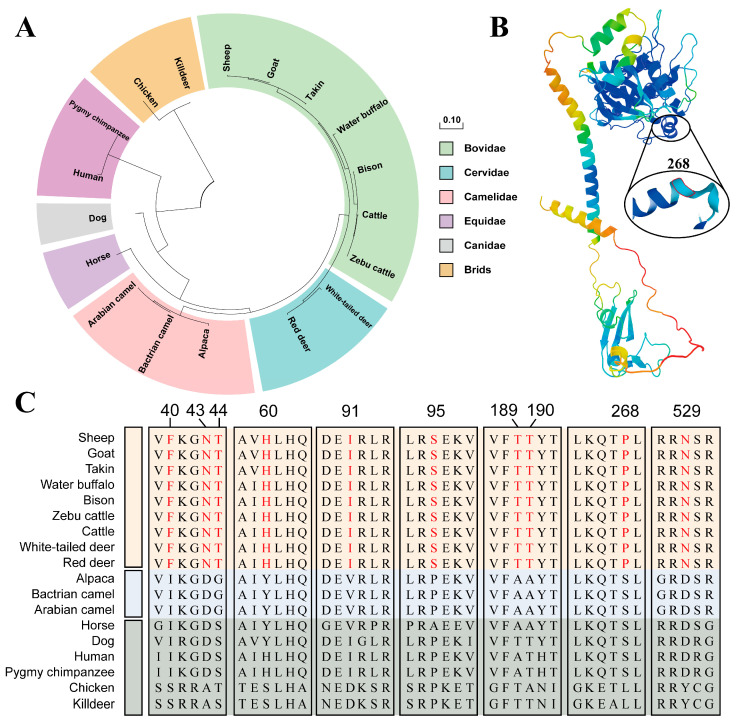
(**A**) Circular evolutionary tree of the BMPR1A protein. (**B**) Three-dimensional (3D) structure of sheep BMPR1A protein. The 3D structure was predicted via AlphaFold2 with a high model confidence level indicated by the color spectrum ranging from yellow to blue. (**C**) The amino acid sites of BMPR1A proteins unique to horned animals. Amino acids marked in red are those that differ between horned and hornless animals.

Figure 3A shows that the evolutionary relationship of proteins was consistent with the evolution of species, and the clustering of horned species in Bovidae and Cervidae suggests that there is similarity in the structure of the BMPR1A protein in these antlered animals in Bovidae and Cervidae. Figure 3C shows the amino acid sites specific to horned animals. Amino acids 40, 43, 44, 60, 91, 95, 189, 190, 268, and 529 were specific to horned animals. These amino acids may play a crucial role in the presence or absence of a sheep horn. Figure 3B shows that amino acid 268 is in the α-helix and has a high predictive accuracy, which may have a significant effect on the presence of a sheep horn.

### 3.4. Allele-Specific Expression in the BMPR1A Gene

**Figure 4:**
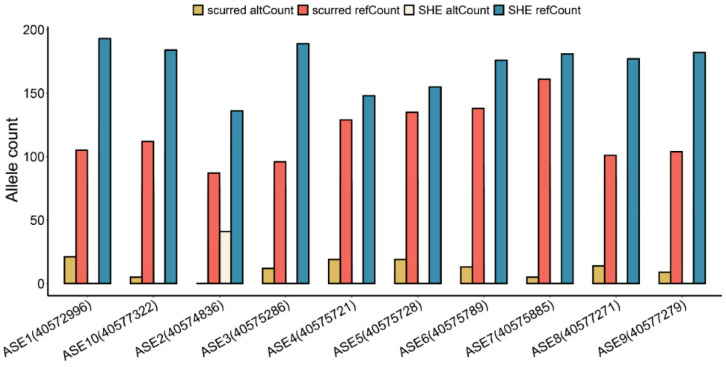
Allele count of 10 ASEs that differed between the scurred and normal horn groups.

Using RNA-seq data, we found 10 allele-specific expressions (ASEs): ASE1 (chr25:40572996), ASE2 (chr25:40574836), ASE3 (chr25:40575286), ASE4 (chr25:40575721), ASE5 (chr25:40575728), ASE6 (chr25:40575789), ASE7 (chr25:40575885), ASE8 (chr25:40577271), ASE9 (chr25:40577279), and ASE10 (chr25:40577322) (Figure 4). ASE2, ASE3, ASE4, ASE5, ASE6, ASE7, ASE8, ASE9, and ASE10 are located on exon 15, and ASE1 is located on exon 12. The alt (alternative) allele Count of ASE1–ASE10 in each of these ASEs were lower than the reference allele Count. These ASEs, except for ASE2, had an alternative count of 0 in the normal horns group. Therefore, these ASE loci may be closely related to horn type. 

### 3.5. Potential Functional Mutations in the BMPR1A Gene

**Figure 5:**
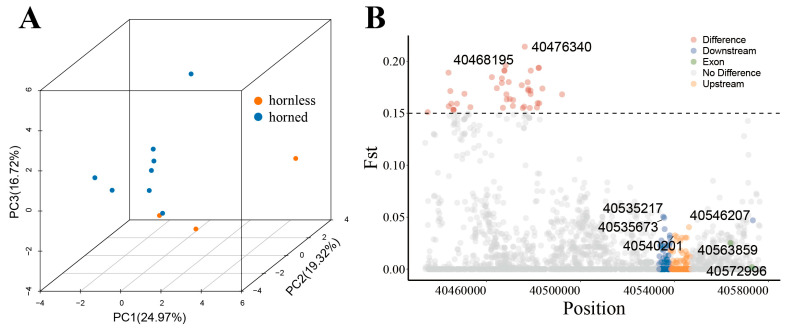
(**A**) Three-dimensional (3D) principal component analysis (PCA) of the *BMPR1A* gene. Each point represents a breed of sheep, categorized as either horned or hornless based on their breed information. (**B**) F-statistics (Fst) of functional loci of the *BMPR1A* gene in horned and hornless populations.

The PCA results showed that these horned breeds of sheep were clearly separated from the polled sheep (Figure 5A). This result indicates that the *BMPR1A* gene can distinguish between horned and hornless populations to some extent and that loci in the region of the gene play a role in regulating horn. We screened a total of 43 loci with Fst values greater than 0.15 and found 18 loci located in exons of this gene (Supplementary Table S3). As shown in Table 1, there are several potential SNP functional loci of *BMPR1A*. These functional loci significantly differed in horned and hornless sheep populations and may play a critical role in sheep horn size.

**Figure 6:**
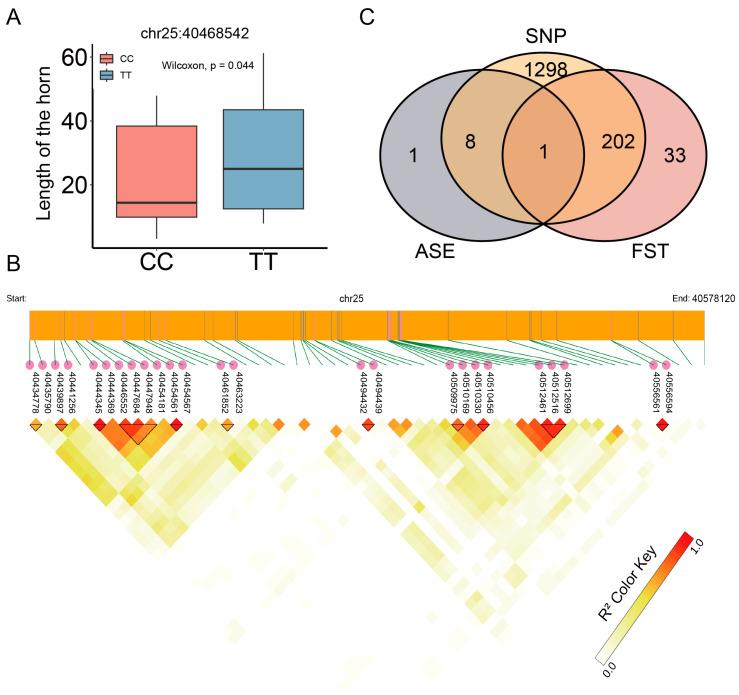
(**A**) Box plots of individual horn lengths for different genotypes, with *p*-values calculated based on the dominant inheritance model and *t*-test. (**B**) LD heatmap of the *BMPR1A* gene. Darker colors represent higher LD values, and black triangles represent LD blocks, which are collections of SNPs with higher LD values. (**C**) Intersection of ASEs, Fst, and SNPs.

As shown in Figure 6A, there was a significant difference in horn length between sheep with different genotypes at chr25:40468542 according to our results under the dominant model. This SNP probably influences sheep horn size and thus horn length. 

We further investigated linkage disequilibrium (LD) among SNPs in the *BMPR1A* gene. We identified 25 SNPs with significant LD based on our LD analysis. Figure 6B shows three SNP sites (40446552, 40447684, and 40447948) in one LD block, whereas another LD block contained three SNP sites (40512461 and 40512516). We examined the intersection between the ASEs, Fst, and SNP locus and determined that the locus 40574836 showed significant differentiation between the populations of the normal horn and scurred groups (Fst = 0.072885). At the same time, within the normal horn group, ASE2 (40574836) exhibited a significantly higher altcount value than the scurred group, which lacked this expression. These findings indicate a strong likelihood that this genetic locus impacts the size of sheep horns. 

## 4. Discussion

In prior research, *RXFP2*, *FOXL2*, *ACAN*, *SFRP2*, *SFRP4*, *WNT3*, and *TNN* were identified as important genes that affect horn phenotype [43,52,53,54]. Although the *RXFP2* gene has been shown to be associated with large horn size in sheep, a study has demonstrated that the horn phenotype of sheep remains unaffected despite partial disruption of the *RXFP2* gene through the use of CRISPR/Cas9 technology [55]. These studies partially explain this phenomenon, indicating that the presence or absence of horns is not solely determined by a single gene or locus. 

The function of the *BMPR1A* gene has been extensively studied. Previous studies of the *BMPR1A* gene have shown its association with bone quality and bone strength and reproduction [56,57]. It has also been shown that the *BMPR1A* gene can also promote adipogenesis [58]. Our analysis revealed discrepancies in the *BMPR1A* gene expression between the normal horn and scurred groups as well as consistency across different sheep breeds. This study did not use qRT-PCR to validate the RNA-seq results. We are aware that this may affect the reliability of our results, and we will add this experiment to future more in-depth studies of the BMPR1A gene on ram’s horn. Using publicly available RNA-seq data, we found that the *BMPR1A* gene is highly expressed in skin and has similar characteristics in both pigs and cattle. This result has also been demonstrated in studies by others [59]. We also found that the expression of the *BMPR1A* gene was higher in ewes than in rams, which is consistent with the results of the study by Gwenn-Aël Carré et al. [60]. Our results are consistent with those of previous studies indicating that this gene is expressed at high levels in the ovaries and uterus of ewes and in the testes of rams [29,30]. Using the ASE results, we successfully pinpointed mutation loci in the normal horn and scurred groups that are likely to influence variation in horn size. It is evident from the results that the *BMPR1A* gene is likely to be located at the amino acid site identified and that sites like ASEs influence *BMPR1A* expression in the skin, subsequently impacting horn size or shape. In a similar study to ours, it was demonstrated that the reversal of the BMPRIA mutation led to a decrease in bone volume and bone formation in mice [17]. We screened 43 SNPs and identified significant differences between the populations of horned and polled sheep. It is probable that these SNPs influenced the size of the sheep’s horn. Using a dominant model and analyzing WGS data, we discovered an association between the g.40468542T>C variant and sheep horn length. Therefore, we consider that g.40468542T>C could possibly affect horn length. Additionally, we conducted an analysis of SNP position chaining in the *BMPR1A* gene region and did not identify any significant SNPs chained to other SNPs. Eventually, an important SNP, g.40574836C>T, was identified. It is both an SNP and exhibits allele-specific expression. 

Multiple studies have demonstrated that *BMPR1A* functions via the transforming growth factor β (TGF-β)/BMP pathway and Wnt signaling in the skin [59,61,62]. According to previous studies, it is proposed that the BMPR1A protein activates SMAD proteins by binding to ligands in the TGF-β signaling pathway [63,64,65]. The Smad2/3/4 signaling pathway in osteoblasts regulates osteogenic differentiation of MC3T3-E1 cells through modulation of ClC-3 chloride channels [66]. The TGF-β family is believed to have arisen during multicellular (metazoan) evolution and is highly conserved [67,68,69,70]. In numerous species, TGF-βs facilitate a range of signaling functions during embryonic and adult stages that regulate tissue-specific differentiation, proliferation, as well as movement of cell-specific or tissue-specific motility [71,72,73]. Members of this family comprise activins, BMPs, growth differentiation factors (GDFs), Müllerian-inhibiting substance (MIS), the nodal, and TGF-βs [73]. TGF-β and BMP signaling pathways played important roles in secondary palate formation in a study of palate development [74]. Chloride-conducting ion channels are present in almost all organisms, including members in every mammalian tissue. These channels play a crucial role in regulating cellular excitability, trans-epithelial transport, cell volume regulation, and intracellular organelle acidification [56]. The function of ClC-3 in bone differentiation may be through the Runx2 gene pathway, which mediates bone formation and remodeling [75]. Xiaolin Lu et al. discovered that changes in ClC-3 chloride channels affect Smad2/3 proteins, demonstrating that the Smad2/3/4 signaling pathway inhibits the regulation of osteogenic differentiation in MC3T3-E1 cells [66]. ClC-3 is expressed in intracellular organelles of osteoclasts and promotes osteoclast bone resorption in vitro via organelle acidification [76]. 

Molecular selection based on the loci identified for horn shape and size offers several advantages for captive sheep breeding. Firstly, it simplifies management as horns can lead to issues during breeding, such as fights between sheep or snagging on fences. Sheep without horns are easier to manage, and the occurrence of these problems can be reduced. Secondly, in intensive farming environments, sheep may injure each other or even the breeder due to their horns. Therefore, sheep without horns reduce the risk of such injuries. Additionally, sheep without horns can use the nutrients that would have been used for horn growth for growth and reproduction, thus improving their survival and reproductive success. This is particularly advantageous in cold regions. For example, Tibetan sheep, which have large horns, may not be as successful in these regions. Finally, animal welfare can be improved by avoiding the painful process of dehorning sheep through selective breeding for hornless sheep.

## 5. Conclusions

The study concludes that there is a correlation between the *BMPR1A* gene and the size and type of horn in ewes. Further analysis indicated that ewes had elevated *BMPR1A* expression levels in skin tissues compared with rams. We identified several potential loci that impact sheep horns, including SNPs and ASEs, along with 10 amino acid sites on the BMPR1A protein that are specifically expressed in horned species. It was found that g.40574836C > T is located on exons, which may have a significant effect on the size of the ewe horns. These loci may significantly impact the size and type of ewe horns and can be used as molecular markers for horn breeding.

## Figures and Tables

**Table 1:** Potential SNP functional loci of *BMPR1A*.

INFO	Mutation Type	Position	Fst	Ref	Alt
SNP1	intron	40476340	0.214027	G	A
SNP2	intron	40468195	0.19581	A	G
SNP3	downstream	40535217	0.0505623	A	G
SNP4	downstream	40535673	0.0494824	C	G
SNP5	upstream	40540201	0.0325271	G	T
SNP6	upstream	40546207	0.040404	T	G
SNP7	exon	40563859	0.0252525	A	G
SNP8	exon	40572996	0.0019107	G	A

## Data Availability

Information about the RNA-seq data in this article can be found at https://www.ncbi.nlm.nih.gov/sra/PRJNA1003277 (accessed on 6 January 2024).

## Supplementary Materials

The following supporting information can be downloaded at: https://www.mdpi.com/article/10.3390/genes15030376/s1, Table S1: Sample Information; Table S2: Mean TPM of BMPR1A gene in different tissues of Cattle, Human, Pig, and Sheep; Table S3: SNP details identified within the BMPR1A gene region.
